# Awareness and Calibration: The Role of Descriptive Norms and Social Desirability in Accurate IAT Score Predictions of Food Items vs. Social Groups

**DOI:** 10.1177/01461672241254447

**Published:** 2024-06-03

**Authors:** Alexandra Goedderz, Adam Hahn

**Affiliations:** 1 German Institute for Development Evaluation, Bonn, Germany; 2 University of Bath, UK

**Keywords:** awareness, attitudes, implicit measures, automaticity, consciousness

## Abstract

Extending research that people are able to predict the patterns of their social group biases on Implicit Association Tests (IATs), we let participants predict and complete IATs toward five different food item pairs and compared the results with the social-groups domain. Participants predicted the patterns of their IAT scores with similar accuracy in both domains, suggesting similar internal awareness (evidenced by comparable within-subjects correlations), even though food evaluations followed less descriptively-normative patterns. At the same time, participants were better-calibrated in communicating their evaluations in the domain of food than social groups (evidenced by higher between-subjects correlations). This discrepancy may partly stem from participants’ tendency to refrain from using harsh labels when predicting social group biases, despite their demonstrated awareness of them: IAT scores predicted as “moderate” or “strong” for food preferences tended to be labeled “mild” for social groups. Discussion centers on the importance of distinguishing between awareness and calibration.

Recent research has documented that people are able to predict the patterns of their scores on several Implicit Association Tests (IATs, Greenwald et al., 1998) toward social groups prospectively (Hahn et al., 2014), contradicting the long-standing hypothesis that the cognitions reflected on implicit measures^1^ are unconscious (Greenwald & Banaji, 1995). Evaluations toward social groups, however, often follow (a) descriptively-normative patterns, and (b) are known to prompt social desirability concerns due to prescriptive norms. This may question if people have true insight into the cognitions reflected on their IAT scores or simply infer them from knowledge about cultural norms, and whether patterns of awareness replicate in domains that are less socially sensitive.

To investigate these factors, we asked participants to predict how they would score on five IATs toward baked goods. We then compared our findings with a comparable sample that completed the paradigm with social groups. If people base their predictions on cultural norms, they should not be able to predict the patterns of their IAT scores in the domain of baked goods, since these may follow less descriptively-normative patterns. In contrast, if people have true insight into the cognitions reflected on implicit measures, they should be similarly able to predict the pattern of their IAT scores in both domains.

The reduced social desirability of the food domain may lead participants to be more willing to use extreme labels in their predictions. Importantly, however, this difference in labeling should not impact participants’ accuracy in predicting the *patterns* of their IAT scores. Instead, it should only impact the accuracy of predicting the strengths of one’s preference compared with other participants. We refer to this difference as a difference between awareness (knowing and reporting one’s relative preferences toward different targets) and calibration (decisions on what to call these preferences, Hahn & Goedderz, 2020). We explain this difference in detail after a review on previous research and theorizing on awareness in the domain of implicit measures.

## Previous Research on Awareness and Implicit Evaluations

Based in part on low correlations between implicit and explicit measures of the same targets (Hofmann et al., 2005), many researchers used to claim that explicit measures assess conscious cognitions while implicit measures tap into unconscious cognitions (Cunningham et al., 2004; Greenwald & Banaji, 1995; The Guardian, 2018; Jost et al., 2002; Nosek, 2007; Phelps et al., 2000; Quillian, 2008; Rudman et al., 1999). However, various dual-process models question this conceptualization and argue that there are other reasons why outcomes on implicit and explicit measures may sometimes diverge (e.g., Fazio, 2007; Gawronski & Bodenhausen, 2006). Based on such dual-process models, Hahn et al. (2014) put the unconsciousness hypothesis to an empirical test. They asked participants to predict how they would score on five IATs toward different social groups (Asian vs. White, Black vs. White, Latino vs. White, Celebrity vs. Regular person, and Child vs. Adult), which they afterwards completed. Results showed that participants were able to predict the patterns of their results on these five IATs accurately (median within-subject correlation between prediction and IAT scores, *r* = .65), suggesting that they had conscious awareness of the evaluations reflected on these IATs (see also Goedderz et al., 2023; Hahn & Gawronski, 2019; Morris & Kurdi, 2023, for replications; and Rahmani Azad et al., 2023, for an extension to gender stereotyping). Importantly, the original studies used highly socially sensitive topics that are matters of continuous public debates: prejudice against minorities and gender stereotypes. There are at least two characteristics of the social-group domain that may limit their generalizability: The existence of descriptively-normative patterns of responses and social sensitivity.

### Descriptive Norms: Knowing One’s Evaluations or One’s Cultural Norms?

Intergroup biases are often discussed in public discourse (e.g., biases toward minority groups and gender stereotypes) and they tend to be culturally shared (Fiske, 2017; Payne et al., 2017). Applied to the topic at hand, people’s ability to predict the patterns of their IAT scores may be less a result of true insight into their own evaluative responses, but rather a demonstration of this cultural knowledge (Morris & Kurdi, 2023). That is, participants in Hahn et al.’s (2014) studies may have simply parroted cultural discussions when they predicted that they will show biases against minority groups and in favor of celebrities and children. To counteract this interpretation, Hahn et al. (2014) showed that their participants could explain their own pattern of preferences better than the preferences of a random other participant, and beyond a binary predictor that contrasted anti-minority bias from pro-celebrity and pro-child bias (see Rahmani Azad et al., 2022, for similar analyses in the domain of gender stereotyping). In this study, we aimed to examine this alternative interpretation more directly by having participants predict nonsocial evaluations that show less consensus, and hence less descriptively-normative patterns, than evaluations of social groups: Evaluations of food items. If it is true that the domain of food shows less consensus, then there should be more between-subjects variation in individual IAT scores (i.e., more variation in how different people evaluate the same targets). However, if people can truly feel the reactions reflected on implicit measures, then participants should still be able to predict the patterns of their IAT scores with similar accuracy, despite the fact that they cannot draw upon cultural knowledge about descriptive norms to the same degree. We further predicted that participants’ own predictions should predict their own IAT results over and above the predictions of other participants in the sample in both domains (Hahn et al., 2014; Rahmani Azad et al., 2022). At the same time, however, if evaluations in the food domain follow less descriptively-normative patterns, other participants’ predictions should be less related to participant’s own patterns of IAT results in the food domain than in the domain of social groups.

Importantly for the present debate: If participants were in fact using these descriptively-normative patterns to make their predictions, they should be less accurate in the food domain than in the social group domain. However, if participants have true insight, they should be similarly accurate in predicting the pattern of their IAT results in both domains. Together, these results would provide evidence for whether people can in fact “feel” the cognitions reflected on implicit measures or whether they predict their patterns based on cultural knowledge.

### Prescriptive Norms: Social Sensitivity in Awareness vs. Calibration

A second distinctive feature of studying evaluative responses toward social groups is that this domain is inherently pervaded with *pre*scriptive norms, resulting in heightened social-desirability concerns: Most people would find it uncomfortable to admit to biases against social groups. On the one hand, this does not seem to have hurt the accuracy with which participants predicted the patterns of their social-group biases in Hahn et al. (2014). On the other hand, however, the authors found relatively lower between-subjects correlations than within-subjects correlations between IAT score predictions and IAT scores.

To understand this difference, it might be worthwhile to dive into the difference between within-subjects and between-subjects analyses of IAT score prediction data. In within-subjects correlations, the accuracy of a person’s prediction is evaluated by comparing it to the *same* person’s other predictions. This relative standing is then compared with the relative standing of their IAT scores. If the ranking and distance between the person’s own predictions of the different targets is the same as the ranking and distance of their IAT scores, the within-subjects correlation will be high. In between-subjects analyses, a person’s prediction is compared with the predictions of *other* people for the same attitude targets. If the ranking and distances between different people’s predictions are similar to the ranking and distances of the same people’s IAT scores, the between-subjects correlation will be high.^2^

Hahn et al. (2014) explained the lower between-subjects correlations in their studies (compared with within-subjects correlations) as a result of different labeling preferences when using the prediction scales for social groups. That is, social desirability concerns may lead participants to abstain from using strong labels (e.g., saying that their reactions are “a lot more positive toward White people”). Importantly, if all participants use the same mild labels to describe their biases (e.g., predicting that their responses will be only “slightly more positive toward White”), then this will restrict the variance of the predictions, and this may explain the low between-subjects accuracy correlations Hahn et al. (2014) found. That is, for a between-subjects correlation to be high, the most biased person in the sample must predict that they will show more bias than other people in the sample. They would have to predict, e.g., that their IAT will show that their implicit attitude is “a lot more positive toward White people”—a potentially threatening prediction. In contrast, for within-subjects correlations to be high, participants need to only predict whether they are more biased against some groups than others (e.g., that they have more positive or negative reactions toward Black people compared with Latinos, Asians, Celebrities and Children). Hence, participants in Hahn et al.’s (2014) studies showed high accuracy in the predictions of their bias *patterns*, even though they were convinced that all of their biases were mild (and milder than the biases of others, see Study 3, Hahn et al., 2014). We refer to this difference between sensing one’s own reactions and calibrating their strengths as the difference between awareness and calibration (Hahn & Goedderz, 2020).^3^

Hahn and Goedderz (2020) have proposed that accurate calibration across a sample of participants depends on two factors: (2a) participants need to have knowledge about conventions for labeling certain reactions, and (2b) they need to be willing to apply these labels to their own reactions.

We propose that in a nonsocial domain like food that is less socially sensitive both of these processes should lead to higher calibration than in socially sensitive domains. First, participants should have more experience in communicating their preferences and hearing other people communicate their preferences for food items than their preferences for social groups. This should lead to more confident knowledge of, e.g., what a “strong preference” means for a food target as opposed to a social group. Second, participants should be more willing to use even strong labels to describe their implicit evaluations of less socially sensitive domains (Process 2b). This should show itself in significantly more extreme predictions for similar IAT scores. As a result, participants should show similar within-subjects, but higher between-subjects correlations between IAT score predictions and IAT scores in the nonsocial domain like food compared with the domain of social groups.

## Implicit Evaluations of Food Targets

Since the invention of the IAT, researchers have used it in a variety of domains such as prejudice and stereotypes (Kurdi et al., 2019), clinical psychology (Nock et al., 2010), personality psychology (Fatfouta & Schröder-Abé, 2018), and consumer choices (Friese et al., 2006). Assessing implicit evaluations of food has become especially popular in health psychology and self-control research as a means to assess individual differences in reactions toward unhealthy food and its consumption (Richetin et al., 2007; Roefs et al., 2006; Seibt et al., 2007). For this article, we chose to study evaluations of baked goods. We did so for several reasons. First, baked goods are culturally important and were in fact declared UNESCO world cultural heritage in Germany (Deutsche UNESCO-Kommission e. V, 2019). Accordingly, we thought it likely that German participants would show strong average IAT preferences, similar to those in the domain of social groups. Second, and more importantly, preferences for baked goods should follow less normative patterns than social groups: Some people prefer simple bread loafs over, e.g., sweet pastry and others prefer sweet pastry over simple bread loafs. Third, and finally, preferences for baked goods are a nonsocially sensitive topic, which should minimize participants’ social desirability concerns when predicting their implicit preferences.

## Overview of the Study

A sample of German university students was asked to indicate their liking for bread rolls (*Brötchen*), croissants (*Croissants*), crispbread (*Knäckebrot*), cake (*Kuchen*), sweet pastry (*Teilchen*), as well as simple bread loafs (*Brot*). Next, they were asked to predict how they would score on a computerized reaction time task measuring their reactions toward the first five categories compared with simple bread loafs. They then continued to complete five IATs toward the same categories.

To compare these results to studies where participants predicted IAT scores toward social groups, we combined samples of German participants who had completed the same social-group paradigm as participants in the studies reported by Hahn et al. (2014) but in German and in the same laboratory as participants in the present baked-goods study. We chose this sample as the most rigorous comparison group as participants from the same population completing tasks in the same language in the same laboratories seemed maximally comparable.

If it is true that people have unique insight into the patterns of their implicit evaluations beyond knowledge about descriptively-normative patterns, participants should show comparably high awareness of their reactions toward baked goods as of their reactions toward social groups, despite the fact that baked-goods preferences follow less descriptively-normative patterns. This would reveal itself in similarly high within-subjects correlations between participants’ predictions for their IAT scores and their actual IAT scores. Our second hypothesis was that participants’ social-group predictions would be less accurate in terms of where they rank in comparison to other participants because they would abstain from using strong prediction labels due to social desirability concerns. If this is true, then data patterns should look different in the less socially sensitive domain of baked goods. First, there should be substantially more extreme predictions for similar IAT scores: More people should openly predict to be “biased” in the domain of baked goods as compared with the domain of social groups. Second, as a result of this more-varied scale usage, participants should show higher between-subjects correlations (but not within-subjects correlations) between IAT score predictions and actual IAT scores for baked goods than social groups.

## Method

All materials, data sets, and analysis files for the analyses reported here are available on the Open Science Framework (OSF) at https://osf.io/8ahbs/?view_only=e9354252dcfe45878dd77c695f6e6bf9. We report all measures and all conditions collected for the baked goods sample run for this article. Selection of data for the social-groups comparison sample is reported below and can be read in its entirety in the respective papers from which it was drawn. The study was not preregistered.

### Baked Goods Sample

#### Participants

We aimed at recruiting at least 100 participants.^4^ One-hundred-and-five participants (85.7% female, 13.3% male, 1 “other,” ages 18–50, median = 22) were approached on campus at the University of Cologne by research assistants and participated in the study for a payment of four Euros or course credit.

#### Materials and Procedure

After signing informed consent, participants began the study by providing explicit evaluations of the baked goods. They indicated how much they “liked” bread rolls (Brötchen), croissants (Croissants), crispbread (Knäckebrot), cake (Kuchen), sweet pastry (Teilchen), and simple bread loafs (Brot), presented in individually randomized orders, on scales ranging from 1 “not at all (überhaupt nicht gerne)” to 7 “very much (sehr gerne).”

Next, participants read an introductory paragraph about the differences between spontaneous affective reactions as opposed to deliberately considered attitudes, similar to the explanations participants have received in the paradigm using social groups (e.g., Hahn & Gawronski, 2019; Hahn et al., 2014). Participants then made a trial prediction for IATs toward cats vs. dogs and flowers vs. insects (they never completed those IATs), before they were told about the IAT attitude pairs they would actually predict and complete in this study. Those were each of the first five categories above contrasted with simple bread loafs (i.e., a “CROISSANT vs. BREAD IAT”).

**Figure 1.**
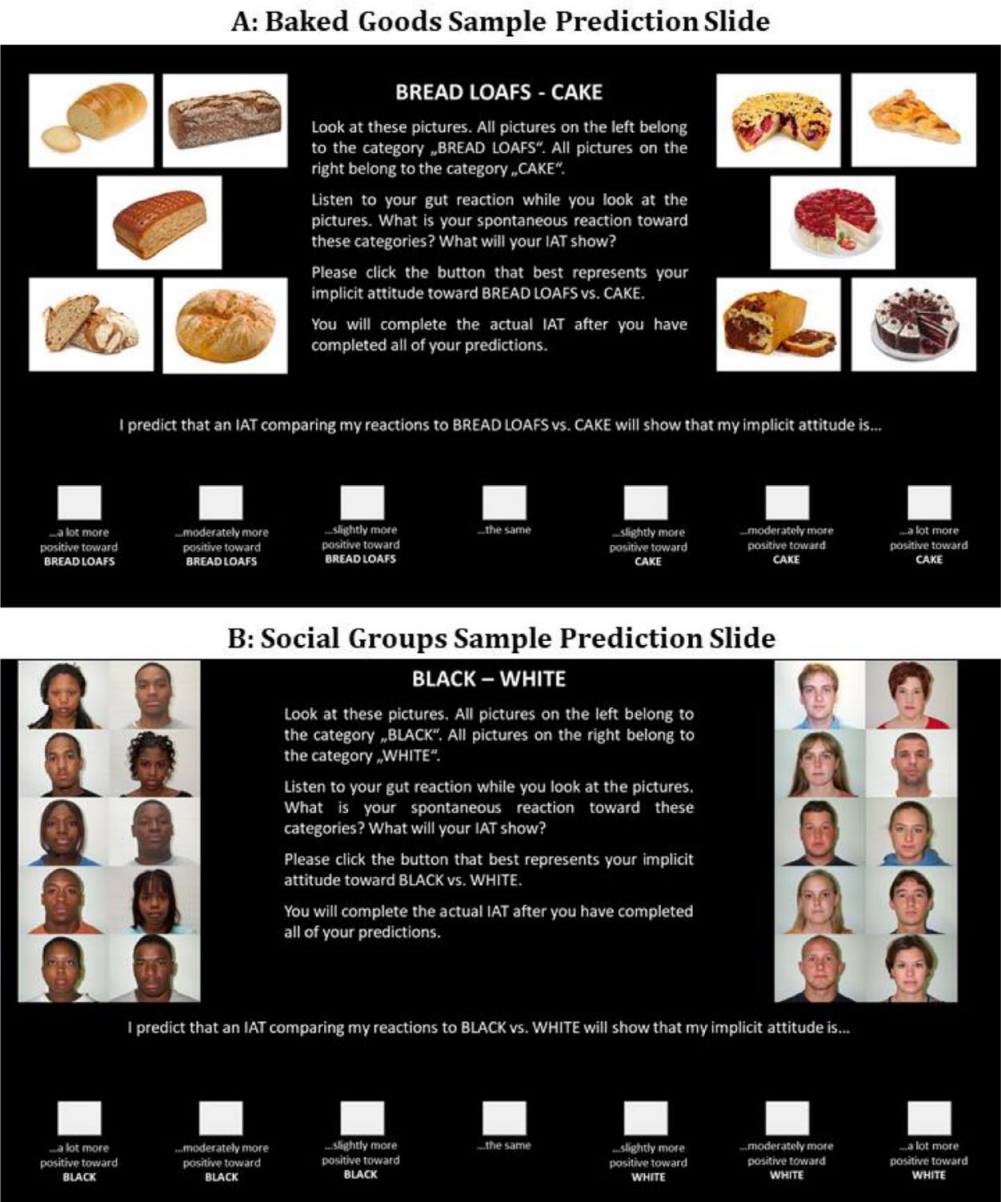
Sample Prediction Slides for the Domain of Baked Goods (Upper Panel) and Social Groups (Lower Panel), Translated to English From German.

The predictions included the five pictures per category that would actually be used on the IATs, grouped together into categories to the left and right of the screens. Participants were asked to predict the spontaneous reaction they would show on an IAT contrasting these two categories on 7-point scales ranging from 1 “a lot more positive towards BREAD” to 7 “a lot more positive towards [the contrast category]” (see Figure 1 for a sample prediction slide).

After completing all predictions, participants completed the five shortened IATs in the manner developed by Hahn et al. (2014). Specifically, participants first completed a 20-trial word-sorting block (words would always be sorted to the same side in all following IATs). Next, they completed the five IATs that each consisted of four blocks: One 20-trial picture-sorting block where participants were trained to sort the pictures only; a 40-trial combined block in which bread was paired with positive words and the contrast category with negative words; another 40-trial picture-sorting block with reversed sorting compared with Block 1; and a final 40-trial combined block with reversed sorting compared with Block 2 (Sorting bread with negative and the contrast category with positive words). An IAT *D*-score was computed using Greenwald et al.’s (2003) scoring procedure such that higher *D*-scores reflect more positive implicit evaluations toward any of the five categories compared with bread (Cronbach’s α: bread rolls = .74, croissants = .73, crisp bread = .72, cake = .69, and sweet pastry = .69). After participants had completed the five IATs, they completed demographic information, were debriefed, and compensated.

### Comparison Social Group Sample

#### Participants

We pooled all participants who had completed Hahn et al.’s (2014) social group paradigm in German in the same laboratory at the University of Cologne under the same conditions. This included a pilot sample of 65 participants (reported in the meta-analysis by Goedderz et al., 2023); 95 and 125 participants from the prediction conditions on Studies 2 and 3 from Hahn and Gawronski (2019); as well as 74 participants from the prediction-with-pictures condition in Hahn and Goedderz (in prep.).^5^ The three samples are analyzed and compared separately in Goedderz et al. (2023). This resulted in a sample of *N* = 359 (78.7% female, ages 17-66, median age 22).^6^ Racial/ethnic identification concerning the categories used in the IATs were not assessed in the pilot sample. Of the 294 participants who were asked, 85.4% indicated that they identified as only White, 7.5% as Middle-Eastern, 0.7% as Black, 1.7% as Latino, 1.0% as Asian,^7^ while 10% indicated they identified with several or yet other categories. One participant did not answer the question.

#### Materials and Procedure

Participants who completed the social-group paradigm completed similar measures as participants in the baked-goods paradigm, with two important differences. First, the paradigm referred to the social categories ASIAN vs. WHITE, BLACK vs. WHITE, LATINO vs. WHITE, CHILD vs. ADULT, and CELEBRITY vs. REGULAR. Second, explicit ratings of these groups were done with thermometer ratings where participants were asked to assess how warm or cool their feelings were toward the groups by choosing a number between 0 and 100 (we opted to use standard 7-point “liking” scales instead of thermometer ratings in the baked-goods study for ecological validity). We used the same 10 stimuli per social group (five male and five female) that Hahn et al. (2014) had used (publicly available at the Minear and Park, 2004, webpage). IAT D-scores were scored such that higher scores reflected a preference for the majority group (White, regular, adult) over the target group (Asian, Black, Latino, child, or celebrity). Otherwise the procedure for the social-groups studies was the same as the baked-goods paradigm. It included (a) explicit ratings in constrained-randomized orders,^8^ (b) explanation of implicit attitudes as reactions contrasted from deliberate attitudes,^9^ (c) five IAT score predictions in randomized orders (see Figure 1 for a sample prediction slide), and finally, (d) five IATs completed in different randomized orders (Cronbach’s α’s: Asian-White = .69, Black-White = .72, Latino-White = .67, celebrity-regular = .58, child-adult = .62). In the studies reported in Hahn and Gawronski (2019), different additional measures followed the IATs not discussed in this article. All studies concluded with demographic information.

## Results

### Strengths of Evaluation

We first tested our assumption that participants had similarly strong reactions toward baked goods as toward social groups. To this end, we ran a mixed-model analysis where participants’ absolute IAT scores were predicted by domain, while domain was allowed to vary both between participants and across different IAT targets. Average absolute IAT scores per IAT are presented in Table 1. They averaged at *D* = .42 in both domains, revealing no effect of domain: *b* = −.001, *SE* = .019, 95% CI = [−.047, .046], *t*(5.45) = −.05, *p* = .961. A nonsignificant random effect of IAT type further suggested that the absolute IAT scores did not vary significantly across the five different IATs within each domain either, IAT-specific random effect: *b* = .001, *SE* = .001, 95% CI = [.000, .006], *Wald Z* = 1.26, *p* = .207. As expected, however, there was significant variation in reactions between participants, as evidenced by a significant person-specific random effect, *b* = .012, *SE* = .002, 95% CI = [.009, .016], *Wald Z* = 6.42, *p* < .001, a point to which we turn next.

**Table 1.** Descriptives and Degree of Calibration: Between-Subjects Correlations Between Predictions and IAT Scores, as Well as Other Relevant Descriptive Statistics for Five Target Pairs of Baked Goods and Social Groups.

Targets	Predictions (7-point scale)		IAT D scores		Absolute IAT D scores		Between-subjects correlations predictions and IAT scores (Calibration)
	*M*	(Between-subjects) Variance	Mean	(Between-subjects) Variance	*M*	*SD*	
Baked Goods							
Bread rolls vs. Bread	5.03	1.87	0.23	0.22	0.42	0.31	.34***
Croissants vs. Bread	4.05	3.76	0.24	0.21	0.41	0.31	.43***
Crispbread vs. Bread	2.43	1.86	−0.09	0.21	0.37	0.29	.31**
Cake vs. Bread	4.78	3.12	0.32	0.21	0.47	0.31	.43***
Sweet Pastry vs. Bread	4.40	3.68	0.33	0.19	0.45	0.31	.46***
**Average**		**2.86**		**0.21**	**0.42**		.39***
Social Groups							
White vs. Asian	4.53	1.04	0.33	0.16	0.42	0.31	.19***
White vs. Black	4.29	0.98	0.36	0.18	0.46	0.31	.19***
White vs. Latino	4.35	0.88	0.31	0.18	0.32	0.23	.26***
Regular vs. celebrity	3.99	1.9	−0.02	0.16	0.47	0.29	.21***
Adult vs. child	2.38	1.43	−0.41	0.13	0.44	0.29	.19***
**Average**		**1.25**		**0.16**	**0.42**		.21***

Preference scores (IAT scores and predictions) in the baked-goods domain mean preference for bread, preference scores in the social group domain mean preferences for the category, White, regular, or adult.

**p < .01. ***p < .001.

### Awareness

#### Baked Goods

To assess awareness of the reactions reflected in implicit evaluations of baked goods, we regressed person-standardized IAT scores onto similarly person-standardized predictions for those IAT scores, separately for each participant on Level 1 of a multi-level model. On Level 2, we looked at the fixed effect to determine the average within-subjects correlation between predictions and IAT scores. Recall that this analysis tests whether participants’ predictions aligned in the same way as their own IAT scores aligned. A high fixed effect would indicate that, on average, every participant’s different predictions showed the same rank order and distances between attitude targets as the same person’s IAT scores. In other words, it assesses whether participants can predict the patterns of their own IAT scores.

Results of this model are presented in Table 2. The fixed effect was *b* = .41, *SE*=.05, 95% CI [.32; .51], *t*(103) = 8.56, *p* < .001. Computing correlations separately for each participant revealed a skewed distribution (Skewness = −.89, *SE* = .24) with the same mean and a median of .59. Fisher-*z*-transformed values showed a mean of *z* = .62, which back-translates to a correlation of .55. In sum, participants were able to predict their pattern of reactions on IATs toward baked goods.

**Table 2.** Awareness: IAT D-Scores Regressed on IAT Score Predictions and Explicit Thermometer Ratings, Simple Relationships and Simultaneous Regressions.

Parameters (DV: IAT *D-score*s)	Baked goods data			Social-groups data		
	Prediction model estimates	Imp.-exp. model estimates	Sim. regr. model estimates	Prediction model estimates	Imp.-exp. model estimates	Sim. regr. model estimates
Fixed effects						
IAT score predictions	.41***		.40***	.52***		.48***
Explicit therm. ratings		.30***	.01		.36***	.09**
Random effect variances						
IAT score predictions	.10**		.08*	.00		.00
Explicit therm. ratings		.09*	.05		.00	.01
Residuals	.59***	.66***	.57***	.58***	.70***	.57***
Goodness of fit						
−2 log likelihood	1,268.38	1,318.32	1,269.62	4,134.23	4,445.33	4,117.76

All variables and the dependent IAT scores are standardized for each individual participant before they are entered in the analysis. Hence, all intercepts are 0 and they are not estimated in these models. Relationships are calculated on standardized scores within-subjects, once per participant, and then aggregated across participants in a multi-level analysis.

* *p* < .05. **p < .01. ***p < .001.

Next, we tested whether the baked-goods data replicated Hahn et al.’s (2014) results that implicit–explicit relations could be entirely explained by participants’ predictions. This would suggest that part of participants’ explicit evaluations is based on their consciously accessible gut reaction (reflected in predictions), but that they consider additional information for their final explicit report.

Results replicated Hahn et al.’s (2014) results. First, the zero-order relationship between explicit evaluations and IAT scores, *b* = .30, *SE* = .05, 95% CI [.21; .40], *t*(104) = 6.20, *p* < .001, was lower than the relationship between predictions and IAT scores (compare Columns 1 and 2 under “baked goods” in Table 2). Second, the relationship between explicit evaluations and IAT scores went to nil when predictions were included in the model, *b* = .01, *SE* = .06, 95% CI [−.11; .13], *t*(184.86) = .18, *p* = .861, whereas prediction accuracy remained unchanged *b* = .40, *SE* = .06, 95% CI [.28; .53], *t*(201.01) = 6.50, *p* < .001.

#### Social Groups

Results from the same analyses conducted on the social-group data set replicated these effects and hence the effects shown in Hahn et al. (2014) in a German context (see right half of Table 2). Within-subject prediction accuracy in the multi-level model was *b* = .52, *SE* = .02, 95% CI [.48; .56], *t*(163.56) = 25.23, *p* < .001. Computing separate correlations per participant revealed a skewed distribution (Skewness = −1.10, *SE* = .13) with the same mean, a median of *r* = .65, and a *z*-transformed mean of *z* = .74, which translates back into a corrected average correlation of *r* = .63. These values thus replicated the values found by Hahn et al. (2014) on a U.S.-American sample on a German sample (.54, .67, and .65, for mean, median, and corrected mean, respectively).

The relationship between explicit evaluations and IAT scores, *b* = .36, *SE* = .02, 95% CI [.32; .41], *t*(1,794,0) = 16.38, *p* < .001, was unexpectedly higher than implicit–explicit correlations found in the literature (Hofmann et al., 2005) and those found by Hahn et al. (2014, both around .20−.29, see also Goedderz et al., 2023). The results nevertheless replicated the pattern reported above. Implicit–explicit correlations dropped when predictions were included in the model, *b* = .09, *SE* = .03, 95% CI [.04; .14], *t*(312.33) = 3.31, *p* =.001, whereas the relationship between predictions and IAT scores remained largely unchanged, *b* = .48, *SE* = .02, 95% CI [.43; .52], *t*(1,765.61) = 19.14, *p* < .001.

### Predictions Beyond Normative Patterns?

A central aim of this study was to examine whether people would be able to predict the patterns of their IAT results in a domain where we expected the patterns to be less descriptively-normative. Specifically, we expected different participants to show more similar evaluations of the same social groups, but more variation in their evaluations of baked goods.

To examine whether reactions toward IATs on social groups followed a more normative pattern than IATs on baked goods, we ran an independent samples *t*-test comparing the mean between-subject variance of the five baked-good IATs with the mean between-subject variance of the five social-group IATs. All individual variances and their mean by domain can be seen in Table 1. In line with our expectations, results showed greater variance in IAT scores for the domain of baked goods (*M* = 0.21, *SD* = 0.01) than for the domain of social groups (*M* = 0.16, *SD* = 0.02), *t*(8) = 4.36, *p* = .002. This suggests that participants showed more similar responses in the social-group domain than in the baked-goods domain.

To then test whether participants’ predictions went beyond these descriptively-normative response patterns, we adapted a procedure employed by Hahn et al. (2014) as introduced by Rahmani Azad et al. (2022): We randomly paired each participant with another participant in the same sample and ran a model in which we predicted every participant’s IAT scores from the randomly-paired other participant. We iterated this process 1,000 times such that we got a distribution of fixed effects that indicated how accurately a randomly paired other participant predicted another participant’s pattern of IAT scores on average. If participants mostly base their predictions on their beliefs about what people would normatively show on such tests, then any participant’s predictions should predict any others participant’s scores as well as participants’ own predictions. Results showed that in both domains, the randomly paired other participants’ predictions predicted participants’ IAT scores above zero in all 1,000 iterations (social-groups sample: *M_b_* = .415, Range_*b*_ [.346; .470]; baked-goods sample: *M_b_* = .202, Range_*b*_ [.058; .321]). This suggests that IAT score patterns follow descriptive norms in both domains; and that they may hence be accurately predicted through knowledge or observation of such norms. Importantly, however, all 1,000 random others’ estimates in the social-groups domain were higher than the random others’ estimates in the baked-goods domain. This is in line with our hypothesis that in the domain of baked goods there may be less of a descriptively-normative pattern than in the domain of social groups.

**Figure 2.**
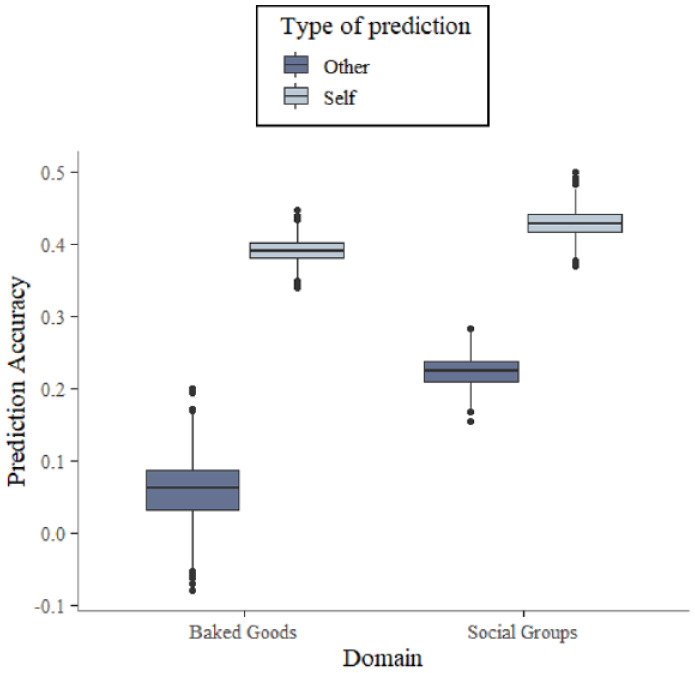
Box plots of Prediction Accuracy Estimates From 1,000 Iterations of a Model Regressing Participants’ IAT Scores Simultaneously Onto Their Own Predictions (Lighter Boxes) and Randomly Paired Other Participants’ Predictions (Darker Boxes) in the Baked-Goods Sample (Left) and the Social-Groups Sample (Right).

To further examine how much variance the participants’ own predictions explained over and above the random others’ predictions, we simultaneously regressed IAT scores onto participants’ own predictions and the randomly paired other’s predictions. Results of the two fixed-effects slopes and their distributions across the 1,000 iterations can be seen in Figure 2. They showed that in both domains, participants’ own prediction outperformed the random others’ prediction in all 1,000 iterations (Social-groups sample: *M_Self_ b* = .428, Range_Self_ *b* [.369; .499], *M_Other_ b* = .223, Range_Other_ *b* [.153; .283]; baked-goods sample: *M_Self_ b* = .391, Range_Self_ *b* [.338; .447], *M_Other_ b* = .058, Range_Other_ *b* [−.080; .200], see Figure 2). This replicates previous findings by Hahn et al. (2014) and Rahmani Azad et al. (2022) and suggests that participants in both domains have unique insight into their implicit evaluations beyond a culturally shared pattern. Importantly, this analysis further showed that the 99% confidence interval of the random other estimates in the baked-goods sample (99% CI = [−.053; .166]) did not overlap with the 99% confidence interval of the random other estimates in the social-groups sample (99% CI = [.171; .276]). This shows that the random other participants’ predictions were less related to participants’ own IAT score patterns in the domain of baked goods than in the domain of social groups. In addition, the 95% confidence interval of the random other estimates in the baked-goods domain still included zero (95% CI = [−024; .136]). This suggests that in the baked-goods domain, the random other prediction may not significantly explain variance of participants’ own patterns of IAT scores above participants’ own predictions in all cases. These findings are compatible with the hypothesis that participants were less able to base their predictions on cultural knowledge about descriptively-normative patterns in the baked-goods domain, and thus predicted their own idiosyncratic patterns instead.

### Calibration

To determine how consistently participants labeled their preferences, we computed the average between-subjects correlation by standardizing IAT scores once for each target pair and regressing this score onto similarly sample-standardized predictions on Level 1 of a multi-level model, and then aggregating the results across target pairs on Level 2 (results are equivalent to computing a simple arithmetic average of the between-subjects correlations between predictions and IATs per target pair).

Recall that between-subjects correlations estimate whether participants’ predictions related to other participants’ predictions in the same way as their corresponding IAT scores relate to those other people’s IAT scores. That is, they effectively estimate whether people who showed more (or less) bias on the IATs than other participants also predicted to show more (or less) bias. As can be seen in Table 1, the average between-subjects accuracy in the baked-goods domain was *b* = .39, *SE* = .04, 95% CI [.31; .47], *t*(524) = 9.74, *p* < .001. The average correlation in the social-groups domain was *b* = .21, *SE* = .02, 95% CI [.16; .25], *t*(1,794.00) = 8.94, *p* < .001.

### Awareness vs. Calibration in Different Domains

Our hypothesis was that people would be better-calibrated when reporting their reactions toward baked goods compared with their reactions toward social groups, despite similar levels of awareness. To test this, we combined the data sets and ran a series of mixed-model analyses.

In a model testing differences in calibration, we regressed sample-standardized IAT scores onto sample-standardized IAT score predictions nested under IAT type, and then looked at the interaction of the predictions with a contrast comparing the baked-goods domain (coded −1) with the social-groups domain (coded 1) on Level 2. This analysis confirmed that between-subject correlations were significantly higher in the domain of baked goods as opposed to the domain of social groups, *b* = −.09, *SE* = .02, 95% CI [−.14; −.05], *t*(2,318.00) = −3.85, *p* < .001. Unexpectedly, another model testing for differences in awareness, where scores were person-standardized and nested under participants, showed that within-subjects correlations between predictions and IAT scores were lower in the baked-goods as opposed to social-groups data, *b* = .05, *SE* = .02, 95% CI [.01; .10], *t*(459.00) = 2.33, *p* = .020.

**Figure 3.**
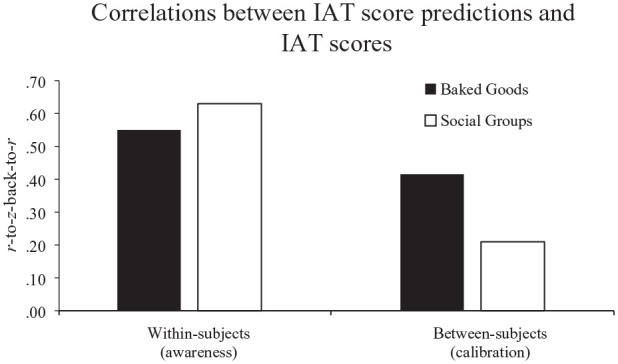
Correlations Between IAT Score Predictions and IAT Scores. *Note*. Individual correlations were Fisher-z-transformed and averaged. The averages are back-transformed to Pearson’s r correlations for easier readability. This back-transformation precludes the usage of error bars.

To account for the skew in the distributions of individual correlations, we also computed each of the individual correlations that went into both of those analyses separately (one per target = 10 in the between-subjects analyses for calibration, one per participant = 464 for the within-subjects analyses for awareness), then *Fisher-z*-transformed them and compared those *z*-scores in *t*-tests accounting for unequal variances (see Figure 3). These analyses continued to show a significant difference in between-subject correlations between the domains, *t*(5.40) = 5.42, *p* = .002, but no significant difference in within-subject correlations, *t*(145.50) = −1.49, *p* = .137. These results are in line with the hypothesis that participants are better-calibrated in their reports for their attitudinal reactions in the domain of food as opposed to the domain of social groups, even though awareness tends to be similar.

### Why Were Participants Better-Calibrated in the Food Domain?

**Figure 4.**
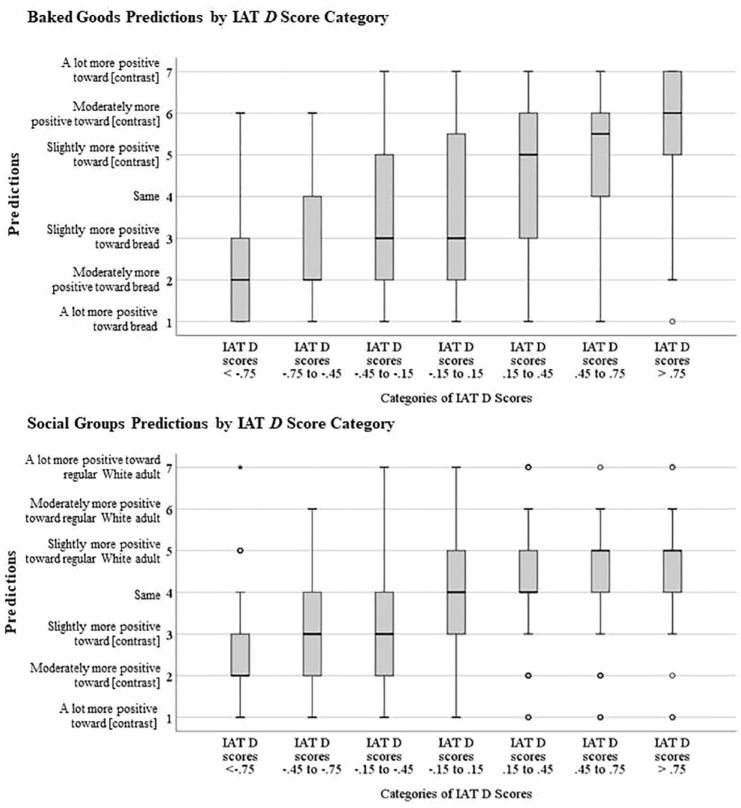
Box plots of Predictions Made for Seven Categories of IAT Scores in the Domain of Baked Goods (Upper Panel) and Social Groups (Lower Panel). *Note*. In the domain of baked goods higher scores reflect more positive evaluation of the contrast categories (bread rolls, croissants, crispbread, cake, or bread) relative to bread. In the domain of social groups higher scores mean more positive evaluations of Whites, adults, or noncelebrities relative to the contrast categories (Blacks, Asians, Latinos, children, or celebrities). Baked goods: *N* = 525 data points, social groups: *N* = 1,795 data points (*N* of D-scores = amount of participants by five IATs).

Recall that we hypothesized that participants would show higher between-subjects correlations (but similar within-subjects correlations) in the domain of baked goods because they would be more reluctant to use harsh labels on the prediction scale for social groups. We ran a series of analyses to test this hypothesis. First, we compared the 10 between-subjects variances in the predictions. Results confirmed that the prediction scales were used with larger variability in the baked-goods (Var = 2.86) as opposed to the social-groups domain (Var = 1.25), *t*(5.54) = 3.51, *p* = .014 (see Table 1). Second, to further examine where this larger variability in predictions came from, we took a closer look at the prediction scale usage. To this end, we categorized all IAT scores into seven categories of equal size increments of .30, ranging from scores below −.75 to scores above .75. We then looked at the predictions participants made on the 7-point prediction scales by IAT scores category, for all IAT *D-* scores in both domains. Box plots of the predictions by IAT score category can be seen in Figure 4. Confirming our expectations, participants used the full 7-point prediction scale to describe their reactions toward baked goods (upper panel), whereas they largely abstained from any labels harsher than “slightly more positive” in the domain of social groups for IAT scores of similar size. *t*-tests accounting for unequal variances confirmed that prediction labels were less extreme for social-group as opposed to baked-good IATs in both the category of IAT scores between .45 and .75, *t*(118) = 3.10, *p* = .002, and the category with scores above .75, *t*(87.38) = 5.12, *p* < .001.

Third and last, we looked into how many participants called their differential reactions to different social groups a preference at all. That is, while participants may willingly describe the patterns of their reactions to social groups, if it is uncomfortable to admit to social-group preferences, participants might be more likely to call their weakest preferences “no preference.” This would result in more “no preference” predictions in the social-group domain compared with the baked-goods domain. To test this idea, we looked at the percentage of IAT scores in each domain that were predicted with a different scale point than 4—the midpoint on the 7-point prediction scale—and compared these numbers to the number of IAT scores that would be categorized as anything but “little to no bias” (conventionally |*D*-scores| > .15).

The number of IATs that had absolute *D*-scores larger than .15 did not differ between domains (baked goods: 77%, social groups: 78%), χ^2^ (1, *N* = 2,320) = .684, *p* = .408, once again confirming that the intensity in reactions on the IATs in the two domains was not significantly different. In line with the idea that participants would be less willing to call their social-group reactions “preferences,” fewer IATs were predicted to show a preference in the social-group domain (71%) than in the baked-goods IATs (91%), χ^2^ (1, *N* = 2,320) = 93.05, *p* < .001.

Once we compared the two categorizations, however, a different picture emerged. Specifically, looking at only those IAT scores that would be classified as “biased” according to IAT score conventions (*N* = 1,820, see Table 3), the number of predictions of these preferences was lower for social groups (56%) than baked goods (67%), χ^2^ (1, *N* = 1,820) = 15.77, *p* < .001. Conversely, looking at IAT *D*-scores that would be classified as no-bias (*N* = 500), this *lack* of bias was more often predicted in the domain of social groups (31%) than in the domain of baked goods (6%), χ^2^ (1, *N* = 500) = 30.94, *p* < .001 (see Table 3). Putting both together, the degree of accuracy in predicting an IAT score bias was similar in both domains (baked goods: 53%, social groups: 50%), χ^2^ (1, *N* = 2,320) = 0.89, *p* = .345.

**Table 3.** Percentage (n) of IAT Score Predictions That Predicted No Preference for an Attitude Target vs. Any Preference, Calculated Separately by Whether the Respective Actual IAT Score Would Be Classified as Biased by Scoring Algorithm From the IAT Webpage.

IAT bias category	Domain	Did not predict IAT preference^1^	Predicted to show IAT preference
“no preference” (|IAT D scores| < .15), *N* = 500	Social groups	**31% (118)**	69% (262)
	Baked goods	**6% (7)**	94% (113)
“preference” (|IAT D scores| > .15), *N* = 1820	Social groups	44% (628)	**56% (787)**
	Baked goods	33% (135)	**67% (270)**

*Note*. Numbers in bold reflect predictions that matched IAT scoring conventions. All differences between domains are significant (see text).^1^ For |IAT *D* scores| > .15, scores that were predicted with a preference in the opposite direction than the IAT showed were grouped with “did not predict IAT preference” to differentiate accurate from inaccurate predictions. Total Number: 525 IAT D scores and predictions for baked goods, 1795 for social groups.

In other words, if IAT scoring conventions (|*D*| > .15) are taken as a benchmark for showing a bias or not, participants more often correctly predicted these biases in the domain of baked goods, but more often correctly predicted the lack of bias by these conventions in the domain of social groups. This resulted in similar accuracy levels between the domains overall.

To summarize, the lack of between-subjects correlations can be explained by the fact that participants abstained from using harsh-sounding labels like “strong” or even “moderate” to describe their preferential reactions in the domain of social groups. They used lower prediction values for all IAT scores above .35 and they more often predicted to show no bias, even when IAT scoring conventions would categorize their reactions as biased. At the same time, however, predictions were similarly accurate in both domains when compared within each participant. That is, within-subjects analyses showed that participants were able to predict the patterns of their social-group preferences accurately, including nuanced differences in reactions to minorities. However, they described these patterns using only labels that sounded mild.

## Discussion

In this study, we investigated whether the findings by Hahn et al. (2014) that people are able to predict the patterns of their IAT scores toward five social groups are generalizable to a different nonsocial attitudinal domain where (a) responses may follow a less descriptively-normative pattern, and that (b) may be less socially sensitive. To this end, we conceptually replicated the paradigm introduced by Hahn et al. (2014) and extended it to the domain of food, specifically baked goods. We then compared the baked-goods sample from the present study to a comparable sample that completed the original social-groups prediction paradigm by Hahn et al. (2014).

Results showed that participants were comparably accurate in predicting the patterns of their IAT results in both domains, although evaluations followed less descriptively-normative patterns in the domains of baked goods compared with social groups. This is in line with the hypothesis that people have unique insight into the cognitions reflected on implicit measures beyond cultural knowledge alone.

We further hypothesized that participants would be better-calibrated in communicating their biased reactions in less socially sensitive topics. Confirming this, between-subjects correlations between predictions and IAT scores were lower in the social-groups domain than in the baked-goods domain, despite similar levels of within-subjects correlations. Confirming the hypothesis that this may be a result of the fact that social desirability concerns influence participants’ usage of the prediction scale, participants used stronger labels to predict their implicit evaluations in the baked-goods domain than in the social-groups domain. They also more often denied showing any bias in the social-groups domain than in the baked-goods domain. We discuss the evidence for each of the points and their implications next.

### Predicting IAT Scores Beyond Normative Patterns

One common explanation for how exactly people came to have accurate knowledge of the patterns they would show on their IATs in Hahn et al.’s (2014) studies is that they simply inferred what scores they would show from cultural knowledge about descriptive norms (e.g., Morris & Kurdi, 2023). Counterarguing this idea, this study showed that participants were able to predict the pattern of their IAT results on baked goods, a domain where one would not expect strong normative patterns. And indeed, in line with the assumption that evaluations toward baked goods would follow less descriptively-normative patterns than evaluations toward social groups, the between-subjects variations on each IAT was larger in the baked-goods domain than in the social-groups domain. Furthermore, the relationship between random other participants’ predictions and participants’ own patterns of IAT results was smaller in the baked-goods domain than in the social-groups domain. And in a simultaneous model, the randomly paired other participants’ predictions in the baked-goods study explained participants’ patterns of IAT results over and above participants’ own predictions in less than 95% of the iterations. Conversely, in the social-groups domain, both the random other and participants’ own predictions jointly explained participants’ patterns of IAT results in all iterations. Together, these findings indicate that the pattern of IAT scores participants produced were less descriptively-normative in the domain of baked goods compared with the domain of social groups.

A test of whether participants were able to predict these nonnormative patterns with the exact same level of accuracy as their social-group patterns yielded mixed results. A direct comparison of raw accuracy correlations suggested that food predictions were less accurate than social-group predictions. In contrast, once the skewed distribution of raw correlations was taken into account via a Fisher-*z*-transformation, this difference disappeared. Although we believe that the analysis on z-transformed values is the more accurate representation, these results remain somewhat ambiguous with respect to whether predictions of food IAT scores are similarly accurate or slightly less accurate than predictions of social-group IATs.

In addition to less normative patterns, another reason food IAT predictions might be less accurate is smaller differences in reactions between targets. It may have been harder for participants to sense the fine nuances between their preferences for, e.g., bread loafs over bread roles than to sense their reactions toward e.g., children as opposed to Black people, because the former vary less. Confirming this idea, the within-person variance for the five IATs was on average lower in the baked-goods study (*M* = .17, *SD* = .14) than in the social-groups sample (*M* = .25, *SD* = .18), *t*(1,041.71) = −11.51, *p* < .001. Future research is needed to confirm these interpretations.

Whether or not the accuracy of predictions was exactly the same or slightly lower for food as opposed to social groups, it is important to remember that participants did predict their patterns accurately in both domains. Their predictions furthermore entirely explained relationships between IAT scores and traditional explicit measures and substantially outperformed the predictions of random other participants. Hence, even if part of the prediction accuracy in Hahn et al. (2014) findings can be explained by replicating normative patterns from descriptive norms, the current studies clearly show that it is possible to predict patterns of IAT scores that aren’t normative and that show large between-subjects variation. Cultural knowledge might help (and the current results might be ambiguous concerning how much it helps), but it doesn’t seem to be a necessary factor to observe one’s own reactions. Future research is needed to tab at exactly which processes lead people to make accurate predictions of the patterns of their IAT scores.

### Predicting IAT Scores in Socially Less Sensitive Domains—Awareness vs. Calibration

One major difference and novelty in the present findings was that participants were less well calibrated in the domain of social groups as opposed to food items. This could be seen in the fact that, even though *within-*subjects correlations between IAT score predictions and IAT scores were comparable in size in both domains, *between*-subjects correlations were substantially higher for baked goods than for social groups.

Analyses further suggested that this difference in between-subjects correlations was due to the fact that participants used only conservative labels to describe their biases toward social groups, whereas they used the prediction scale much more liberally when predicting their IAT scores toward baked goods. The same IAT scores that were labeled “moderate” (IAT *D* scores between .45 and .75) or “strong” (IAT *D* scores larger than .75) in the domain of baked goods were labeled predominantly as “mild” in the domain of social groups. In addition, analyses of whether participants would describe their reactions as preferential showed that more IAT scores that would be classified as biased by IAT scoring conventions were predicted as not biased in the social-group domain compared with the baked-goods domain, even though the proportion of such biased IAT scores was the same in the two domains. Importantly, however, these mild labels sufficed to describe patterns of IAT scores in the social-groups domain, confirming that participants were generally aware of their reactions, even if unwilling to name those reactions anything but “mild,” or preferential at all. This unwillingness to use harsh-sounding descriptors in socially sensitive topics may be a main reason for the low levels of calibration in the domain of social groups.

Specifically, Hahn and Goedderz (2020) have posited that awareness versus calibration of automatic cognitions depend on different psychological processes. Awareness depends on internal processes, specifically (1a) the strength of a signal a process produces and (1b) whether a person pays attention to said signal. Calibration, however, cannot depend on internal processes. Whether or not one’s reaction is stronger or weaker than the reactions of other people is information that does not reside in a person’s own sensory system and cannot be learned via introspection. Instead, calibration should depend on (2a) whether a person knows the social conventions of what a certain reaction is labeled by the comparison sample (e.g., what a “slight” vs. “strong” preference feels like, what other people would say), as well as (2b) willingness to apply these labels to one’s own cognitions (e.g., willingness to say “I have a strong preference for Group A”).

Concerning awareness, analyses confirmed that participants showed similarly strong reactions toward food as toward social groups (process 1a was constant), and all participants were asked to look at pictures and pay attention to their own reactions, such that attention was also held constant across the domains (process 1b). On the one hand, the current data are compatible with this model since both processes are met in both domains and we found similar awareness in both domains. On the other hand, the current study did not show whether participants truly “felt” their reactions because they were asked to pay attention to them, or whether they used other processes, such as inferences about what they think they should likely show. As the discussion above shows, predicting the patterns of one’s score seemed to possibly be somewhat easier when those patterns follow cultural norms. This suggests that people may be using other information to predict their IAT scores in addition to sensing their own reactions (Morris & Kurdi, 2023). Future research is needed to fully understand which information goes into IAT score predictions.

Concerning calibration, we have so-far focused our discussion on process (2b); the fact that people should be much more willing to call their food preferences than their social-group preferences “strong.” And indeed, the data analyses we presented are compatible with the interpretation that people are unwilling to apply certain bias labels to their social-group biases (Hahn & Goedderz, 2020). However, we believe process 2a may be at play here, too. Talking about one’s food preferences and seeing associated behavior (e.g., how much a person likes and eats something) is much more common than talking about social-group preferences. As a result, people should have much more knowledge about what a “mild” vs. “strong” food preference feels like than how one would refer to a bias against a social group. While this interpretation remains speculative, it is compatible with the data. Future research is needed to validate other aspects of Hahn and Goedderz’s (2020) model.

The distinction between awareness and calibration maps onto the process of noting a sensory reaction and mapping this reaction onto a scale, which has a long tradition in sensory perception research (Lim, 2011). We apply it to the domain of predicting one’s IAT scores because the distinction seems especially pertinent when applied to a domain where (2a) talking about one’s reactions is uncommon and (2b) certain labels for one’s reactions are less socially desirable than others. Some readers may wonder, however, whether this distinction maps on as cleanly onto within- vs. between-subjects analyses as we have argued so far, or whether other measurement differences could also explain the results we found. For instance, the size of correlations critically depends on how much variance there is in each of the variables. Since our homogeneous student samples showed less between-subjects variation in their reactions toward social groups than baked goods, the low between-subjects correlations might simply be a result of the fact that there was little variance to predict.

We ran a variety of analyses to test which aspects of the data could best explain the lower between-subjects correlations for social groups as opposed to baked goods. Those showed that it is primarily the reduction in variance in the usage of the prediction scale, not the reduction in variance on the IAT scores, that explains the lower between-subjects correlations in the social-group domain. That is, Figure 4 visually demonstrates that the reduction in variance in IAT scores (from .21 for baked goods to .16 for social groups, a reduction of 24%) was far exceeded by the reduction in the variance of the prediction scale (2.86% to 1.25, 57% reduction). In other words, while IAT *D*-scores still spanned the whole spectrum from −1 to 1 in both domains, participants used the prediction scale in the domain of social groups essentially only between scale anchors 3 and 5, even though their absolute reactions toward the targets were virtually identically strong in the two domains. Hence, while both within- and between-subjects correlations can always be influenced by a variety of factors, the current data indicate that in this particular case, participants’ unwillingness to call their biases anything but mild may be the most important reason that between-subjects accuracy correlations were lower for social groups than baked goods.

On a more abstract level, it is important to note that people do talk about their attitudes all the time. From food to abstract political convictions, to music, clothes, and any conceivable attitude targets, humans evaluate things constantly, and they share these evaluations too. Processes 2a (knowledge of conventions) and 2b (willingness to apply labels to one’s preferences) should thus generally be high for most attitude targets in any given culture. That is, social groups might be the outlier domain rather than the norm in people’s ability and unwillingness to describe their preferences. Future research is needed to extend the current findings to other attitude targets.

One question the current data pose is weather participants can be taught to calibrate their IAT score predictions to be consistent with each other in the domain of social groups. While this is beyond the scope of this article, in principle both processes 2a and 2b are learnable. In fact, encouraging people to entertain that social-group biases that look mild from one perspective can look strong from another may motivate them to control their biases in future behavior (Forscher et al., 2017). Hence, targeting calibration could be a promising avenue for intervention work. Future research is needed to investigate how learnable and malleable social calibration processes are and whether improving them may have positive consequences.

In sum, our data suggest that even though participants were similarly aware of their differential reactions toward different social groups as of their differential reactions toward food items, they described the same preferences as milder in the domain of social groups as opposed to the domain of food. As a result, within-subjects correlations between predictions and IAT scores were similar in both domains, but between-subjects correlations were lower for social groups as opposed to baked goods.

### Limitations

The present study focused on two domains of attitudes that we felt differed maximally in terms of social-desirability concerns and normative patterns. However, these domains do of course differ on countless other dimensions, too, and there are countless additional attitude domains that may fall anywhere on these dimensions. As such, this study can only be viewed as one incremental step toward understanding the different processes that factor into awareness and calibration. In addition, we limited our implicit measure to the IAT to stay as close to the original paradigm as possible. Morris and Kurdi (2023) recently provided first evidence that the effect of awareness of implicit attitudes generalizes to other implicit measures such as the Affect Misattribution Procedure (AMP, Payne et al., 2005) and to 57 different broadly and randomly sampled attitude targets. These findings make us optimistic that our assumptions may apply more broadly to the cognitions reflected in implicit evaluations independent of the measure used to capture these cognitions, and that it extends to many more attitude targets. Future systematic comparisons of attitude targets from different domains are necessary to confirm our theoretical interpretations.

Another major limitation of this project is that we compared two independent samples in a quasi-experimental design rather than randomly assigning participants to conditions in the same study. This potentially invites the question of whether the differences we found (mainly on calibration) are really a result of domain or whether our baked-goods sample was simply better-calibrated than our social-groups sample. While lack of random assignment makes this a theoretical possibility, we carefully selected a comparison sample across all our available data sets that was drawn from the same student population in the same lab. In addition, the only one difference we found is theoretically consistent with our theorizing: People showed similar awareness but differential calibration. Hence, we believe our results can be attributed to true differences between the domains and not random differences between the samples, despite the quasi-experimental nature of our design. Future research is needed to confirm these points.

## Conclusion

The purpose of this article was to extend Hahn et al.’s (2014) findings that people can predict the patterns of their IAT scores toward social groups to a domain that is nonsocial, tends to show less descriptively-normative patterns, and where there are fewer concerns with social desirability. To meet these goals, we chose the domain of food items, specifically baked goods. This study replicated findings by Hahn et al. (2014) in the domain of baked goods and showed that participants were able to accurately predict the patterns of their IAT scores toward baked goods even though the reactions toward these targets followed less descriptively-normative patterns. These findings support the notion that the cognitions underlying implicit measures can be consciously perceived rather than just inferred; and that implicit measures do not capture attitudes people are “unable to report.” Instead, they are more compatible with the notion that implicit measures capture spontaneous reactions that may sometimes evade attention, but that can be observed when a person is encouraged to pay attention to their spontaneous reactions.

In contrast to similar levels of awareness, participants differed in their level of calibration between the domains. They freely chose more extreme labels to describe their food preferences than their social-group preferences, and these more extreme labels were better-aligned between participants. These findings suggest that people may often be aware but miscalibrated in their biases toward social groups. Most importantly, they suggest that distinguishing awareness from calibration might be important if one wants to understand what people know and don’t know about their own cognitions.

## Notes

1. In line with de Houwer et al. (2009), we use the term “implicit measures” to refer to measurement outcomes (in this case evaluations) that are inferred from instruments that limit a participant’s ability to control the measurement outcome. This term is contrasted from the term “explicit measures” which we use to describe measures that are (explicitly) reported under full control in self-report measures. Hence, we do not mean to imply that the underlying cognitions reflected on implicit vs. explicit measures are necessarily different. We are trying to contribute to understanding what those differences, if any, may be.

2. The size of both within- and between-subjects correlations are of course also influenced by other factors than those described here. We focus on one important theoretical distinction here, but we will return to this point in the discussion.

3. The difference between awareness and calibration maps onto a long-standing methodological distinction between “sensory processes” and “cognitive processes” in rating one’s evaluations and other sensory perceptions (e.g., Lim, 2011): A person needs to first decide what the sensory perception is and then how to translate it onto a rating scale.

4. The effect sizes for accuracy correlations reported by Hahn et al. (2014) are quite large, a correlation of .54 necessitates only 47 participants to show a significant relationship with a power of 99% according to the GPower program (Faul et al., 2007). We opted for a larger number because we did not know which level of accuracy participants would show in this paradigm, and to have sufficient power to detect differences between the baked-goods and social-groups data.

5. Participants in the other conditions of Studies 2 and 3 in Hahn and Gawronski (2019) did not predict their IAT scores. The participants from Hahn and Goedderz (in prep.) who were not included either predicted their IAT scores without pictures, or simply indicated their spontaneous reactions with no announcement of a test measuring said reactions. We only included the 74 participants who completed the exact same prediction task with pictures as all other participants included here.

6. No participants were excluded in the pilot sample. Exclusions in the samples drawn from Hahn and Gawronski (2019) and Hahn and Goedderz (in prep.) can be seen in the respective papers. Data were never analyzed before these exclusions were made.

7. Participants who identified as both White and a racial minority category were grouped with the minority category.

8. Three blocks of groups were always rated together, but their order randomized: (a) the racial groups in random orders, (b) adults and children, and (c) celebrities and regular people.

9. The explanations varied slightly between the studies. However, these differences did not produce consistent differences, such that they are combined here (see Hahn et al., 2014).
